# The “specific” P-glycoprotein inhibitor zosuquidar (LY335979) also weakly inhibits human organic cation transporters

**DOI:** 10.1007/s00210-024-03743-y

**Published:** 2024-12-24

**Authors:** Gzona Bajraktari-Sylejmani, Rajamanikkam Kamaraj, Dirk Theile, Petr Pávek, Johanna Weiss

**Affiliations:** 1 Internal Medicine IX - Department of Clinical Pharmacology and Pharmacoepidemiology, Heidelberg University, Medical Faculty Heidelberg, Heidelberg University Hospital, Im Neuenheimer Feld 410, 69120 Heidelberg, Germany; 2 Department of Pharmacology and Toxicology, Faculty of Pharmacy, Charles University in Prague, Heyrovskeho 1203, 50005 Hradec Kralove, Czech Republic

**Keywords:** Zosuquidar, LY335979, Metformin, P-glycoprotein, Organic cation transporters, Molecular docking

## Abstract

Zosuquidar (LY335979) is a widely used experimental P-glycoprotein (P-gp) inhibitor, which is commended as very potent but also as very specific for P-gp. In this in vitro and in silico study, we demonstrated for the first time that zosuquidar also inhibits organic cation transporters (OCT) 1–3, albeit less potently than P-gp. This still has to be kept in mind when zosuquidar is used to inhibit cellular efflux of P-gp substrates that are concurrently transported into the cells by OCTs. To avoid interference in these assays, zosuquidar concentrations should be kept below 1 µM.

## Introduction

Zosuquidar (LY335979, formerly RS-33295–128) has been developed as a third-generation P-glycoprotein (P-gp) modulator (Slate et al. 1995; Dantzig et al. 1996; Starling et al. 1997; Lai et al. 2020). Compared to first-generation inhibitors like verapamil or quinidine, it has multiple advantages like higher binding affinity, high specificity, lack of pharmacokinetic interactions and a high therapeutic index (Starling et al. 1997; Dantzig et al. 1999; Shepard et al. 2003).

Zosuquidar, in fact, has been tested in several clinical studies as a chemosensitiser, but its efficacy was not convincing, although it might be improved by changing infusion strategies and patient stratification (Lai et al. 2020; Marcelletti and Sikic 2024). Altogether, the clinical development of zosuquidar is no longer promoted, but it still has a high significance and widespread usage as an experimental P-gp inhibitor.

The high P-gp inhibitory potency of zosuquidar is reflected by its low IC_50_, which is in the nanomolar range, depending on the substrate and the cell model used (Slapak et al. 2001; Green et al. 2001; Weiss and Haefeli 2006; Ozgür et al. 2018). So far, zosuquidar is considered a specific inhibitor for P-gp. Other ATP-binding cassette (ABC-) transporters such as multidrug resistance-associated proteins (MRP) 1 and 2, and breast cancer resistance protein (BCRP) are not affected by this compound (Dantzig et al. 1999; Shepard et al. 2003). Among the uptake transporters, only inhibition of the organic anion transporting polypeptide 1A2 (OATP1A2) with an IC_50_ of 6.7 µM has been demonstrated (Bakos et al. 2020). However, we recently obtained some experimental indications that zosuquidar likely also inhibits organic cation transporters (OCTs) (Bajraktari-Sylejmani et al. unpublished results). This additional OCT inhibition could considerably influence cellular drug uptake/efflux experiments and thus data interpretation. For instance, when zosuquidar is used to inhibit cellular efflux of P-gp substrates whose OCT-mediated uptake is concurrently inhibited as well, the observed diminished drug uptake can falsely be interpreted as a poor P-gp inhibition effect. Accordingly, we scrutinised in vitro and via docking analyses whether zosuquidar can inhibit these important uptake transporters.

## Material and methods

### Materials

Cell culture medium DMEM, foetal calf serum (FCS), supplements, Hank’s buffered salt solution (HBSS), phosphate buffered saline (PBS), the Cytotoxicity Detection Kit (LDH), Casy® ton, Casy® clean, and zosuquidar hydrochloride were obtained from Sigma-Aldrich (Taufkirchen, Germany). Dimethyl sulfoxide (DMSO), geneticin (G418) and crystal violet were from AppliChem (Darmstadt, Germany). Purified water was produced using an arium® mini (Sartorius, Göttingen, Germany) ultrapure water system.

### Stock solutions

Stock solution of zosuquidar was prepared in DMSO (10 mM) and of metformin in aqua bidest. All stock solutions were stored in aliquots at −20 °C.

### Cell culture

HEK293 (available at ATCC, Manassas, VA, U.S.A.), HEK293-OCT2, and HEK293-OCT3 cells were cultured in DMEM with 10% FCS, 2 mM glutamine, 100 U penicillin/100 μg streptomycin, and 600 µg/ml geneticin (except for HEK293) under standard cell culture conditions. The overexpressing cell lines were a kind gift of Herrmann Koepsell and were generated and characterised as described by Lee and coworkers (Lee et al. 2009). HEK293-Flp-In-OCT1 cells were a kind gift of Lukas Gebauer (Göttingen University, Göttingen, Germany) and generated and characterised earlier (Saadatmand et al. 2012; Seitz et al. 2015). They were cultured under standard cell culture conditions in DMEM with pyruvate, with 10% FCS, 2 mM glutamine, and 100 U penicillin/100 μg streptomycin sulphate.

### Cytotoxicity assay

To exclude possible influences of zosuquidar on cell viability, which could influence the results of the inhibition assays, zosuquidar was tested for possible short-term cytotoxic effects in all HEK293 cell lines by means of the Cytotoxicity Detection Kit according to the manufacturer’s instructions. The assay quantifies the activity of the lactate dehydrogenase (being released from damaged cells into the supernatant) and was used to evaluate cytotoxicity after 2 h of incubation with the test compounds. Zosuquidar was tested in four concentrations (semilogarithmic dilutions) up to the highest soluble concentration of 50 µM and each concentration was tested in quadruplet.

### Inhibition of metformin uptake in OCT over-expressing cell lines by zosuquidar

The OCT inhibition assay was established and validated previously (Bajraktari-Sylejmani et al. 2024). Zosuquidar was tested for its ability to inhibit the OCT-mediated transport of metformin into HEK293 cells overexpressing either OCT1, OCT2, or OCT3. Cells were used in suspension to enable adjustment of the cells to a constant number in each sample and to allow measuring their exact volume (Bajraktari-Sylejmani et al. 2024). Since the inhibitory potency might be underestimated when substrate and inhibitor are applied concomitantly (Tátrai et al. 2019; Nozaki and Izumi 2023), a pre-incubation approach was chosen. In brief, cells were washed once after detachment and their volume was measured in a CASY® cell counter (OMNI Life Science GmbH + Co KG, Bremen, Germany). For each incubation point replicate, 1 × 10^6^ cells were pre-incubated in low-binding 1.5 ml tubes for 5 min in 100 µl HHBSS at 37 °C on a rotary shaker. Afterwards, either HHBSS alone (control) or zosuquidar solution (final concentration 0.1–50 µM) was added and the cells were incubated for 20 min at 37 °C on a rotary shaker. After adding metformin (final concentration 10 µM), cells were further incubated for 10 min (HEK293-Flp-In-OCT1 and HEK293-OCT3) or 2 min (HEK293-OCT2) at 37 °C on a rotary shaker. Cells were pelleted by centrifugation at 4 °C and washed twice with ice-cold HHBSS before freezing the pellet at – 20 °C until extraction. Sample extraction and quantification of metformin by ultra-performance liquid chromatography coupled to mass spectrometry (UPLC-MS/MS) was conducted as described in detail previously (Bajraktari-Sylejmani et al. 2024). Each experiment was conducted with technical triplicates and repeated 3–6 times. Zosuquidar could only be tested up to 50 µM, because higher concentrations were not soluble in HHBSS.

### In silico molecular docking

Docking studies were prepared using the Schrödinger Maestro 13.3 software package. The ligand structures were drawn with ChemDraw 22.2. For ligand preparation, the conformation of compounds was generated and energy-minimised using the LigPrep tool using force field OPLS4.

The 3D crystal structures of the inhibitor-binding organic cation transporters OCT1 and OCT3 were obtained from the RCSB Protein Data Bank with PDB codes 8ET8 and 7ZH6, respectively. The modelled structure of OCT2 was retrieved from AlphaFold with the identifier AF-O15244-F1 (Uniport ID: O15244) (Varadi et al. 2023). The inhibitor binding site of OCT2 was predicted using the SiteMap tool within Schrödinger’s Maestro suite, which also confirmed the presence of tyrosine residues (Y362 and Y377), known to be critical phosphotyrosine sites for inhibition (Sprowl et al. 2016).

The OCT transporters were prepared using the Protein Preparation Workflow, which involved optimisation, charge calculation, deletion of a cocrystal ligand, and addition of hydrogen, among other steps. Docking of ligands at inhibitor binding sites was performed using the GLIDE XP tool to ensure a high degree of accuracy (Friesner et al. 2006). We used various known inhibitors as controls and performed redocking as necessary to confirm the poses. The proposed binding modes were visualised and analysed using a combination of software tools. The LigPlot + v.2.2 was used to generate ligand interaction diagrams in 2D (Laskowski and Swindells 2011), while PyMOL 3.0.2 or ChimeraX v1.4 was used to produce 3D images (Phondeth et al. 2024; Brožová et al 2023; Meng et al. 2023.

### Statistical analysis

Data were analysed using GraphPad Prism Version 10.1.0. (GraphPad Software, San Diego, CA, USA). IC_50_ values were calculated using the four-parameter fit (sigmoidal dose–response curves with variable slope). For the OCT1 inhibition curve no constraints were set for top and bottom. For the OCT2 and OCT3 inhibition curves, the bottom was set to zero, because no clear inflection point was achieved. For testing whether the effects observed for zosuquidar were statistically significant, ANOVA was used with Dunnett’s multiple comparison test for post hoc pairwise comparison with the control without inhibitor using GraphPad InStat version 3.05 (GraphPad Software, San Diego, CA, USA). A p-value of 0.05 was considered significant.

## Results

**Fig. 1:**
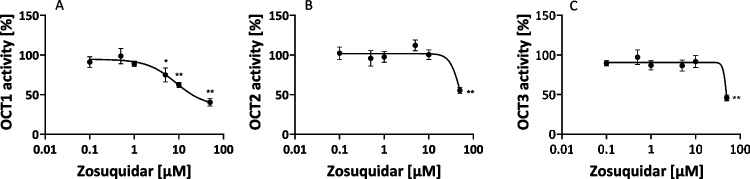
Inhibition of metformin transport in OCT over-expressing cells by zosuquidar. Depicted is the mean ± S.E.M of 4 biological replicates (OCT1, A), 6 biological replicates (OCT2, B), and 3 biological replicates (OCT3, C) with technical triplicates for each value. *P*-values were determined by using ANOVA with Dunnett’s multiple comparison test for post hoc pairwise comparison with the control without yohimbine. * *p* < 0.05, ** *p* < 0.01

The in vitro assay using metformin as an OCT substrate in HEK293 cells overexpressing the corresponding OCT revealed that zosuquidar is an inhibitor of OCT1-3 (Fig. 1). However, OCT2 and OCT3 were only inhibited at the concentration of 50 µM. Due to its low solubility, zosuquidar could not be tested at concentrations higher than 50 µM and thus, no complete sigmoidal inhibition curves and no IC_50_ values for OCT2 and OCT3 could be calculated. In contrast, OCT1 was already inhibited at concentration ≥ 5 µM and an IC_50_ value of 7.5 ± 3.7 µM could be calculated. Cytotoxicity assay demonstrated that zosuquidar was not toxic up to the maximum concentration tested (50 µM).

**Fig. 2:**
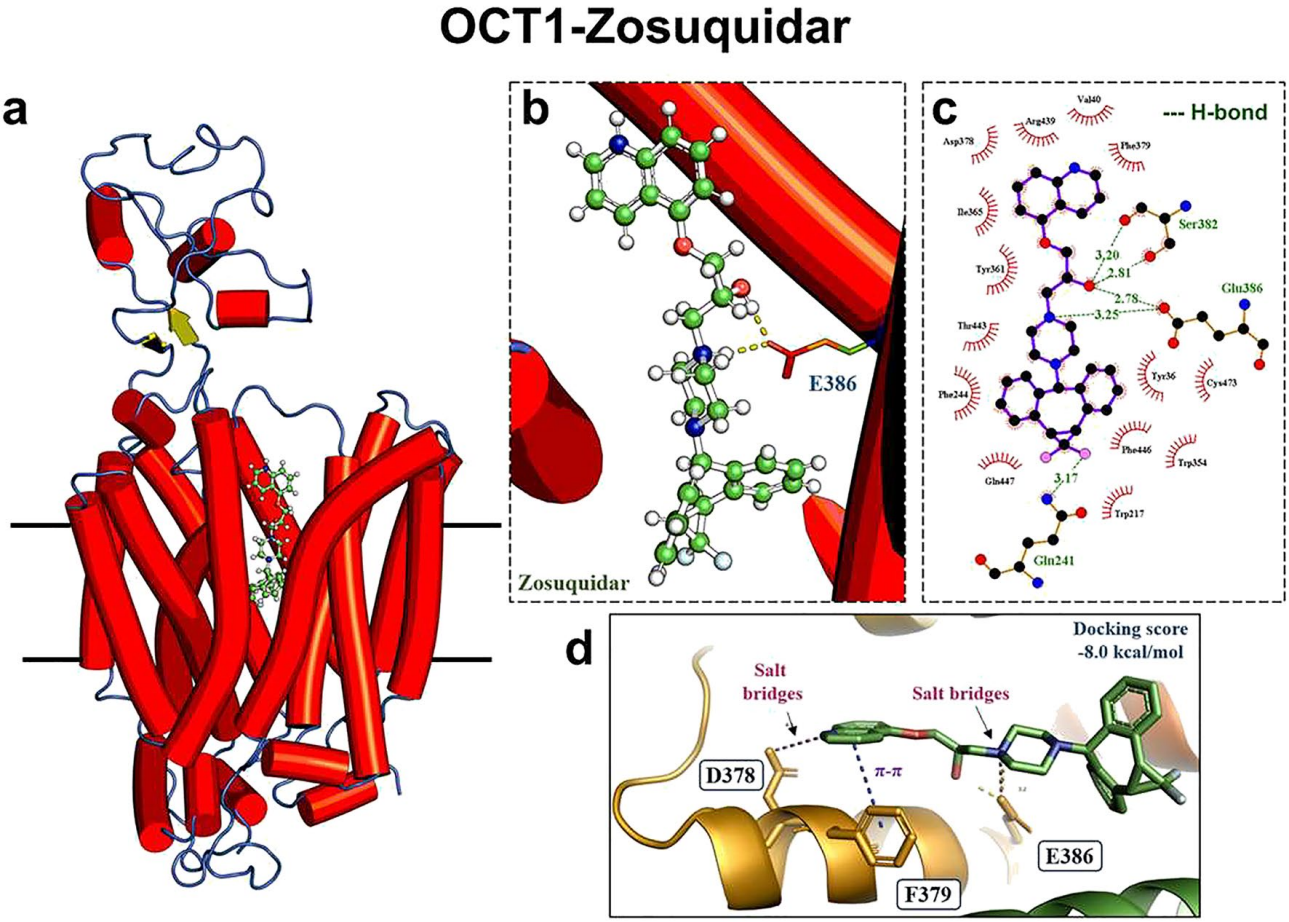
Molecular interaction of zosuquidar with the OCT1 protein. **a** Overall structure of OCT1 with zosuquidar bound within the binding pocket. **b** Close-up view of the drug-protein interaction, with key residues such as E386 interacting via hydrogen bonds, depicted by yellow lines. **c** Detailed 2D interaction diagram between zosuquidar and surrounding amino acids, highlighting hydrogen and hydrophobic interactions. **d** Focus on specific salt bridge and pi-pi interactions with the corresponding docking scores

The OCT1-inhibitory effect of zosuquidar was underlined by molecular docking simulations. The analyses revealed strong double hydrogen bond interactions with S382 and the acidic residue E386 of OCT1 (Fig. 2c). Additionally, the difluorocyclopropane group in zosuquidar formed hydrogen bonding interactions with Q241, similar to the inhibition mechanism of OCT1 by spironolactone, which has been described earlier (Fig. 2c) (Zhang et al. 2024). Other non-covalent interactions included pi-pi interactions with F379 and salt bridges with E386 and D378 residues (Fig. 2d). The docking score was calculated as −8.0 kcal/mol (Table 1).

**Table 1:**
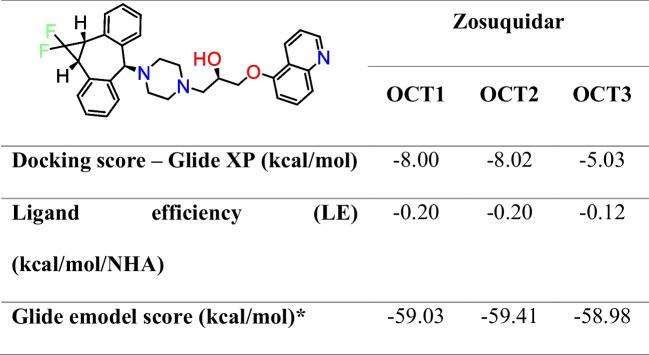
GLIDE XP docking of zosuquidar to the OCT1-3 inhibitor site

^*^emodel = electrostatic and van der Waals energies

**Fig. 3:**
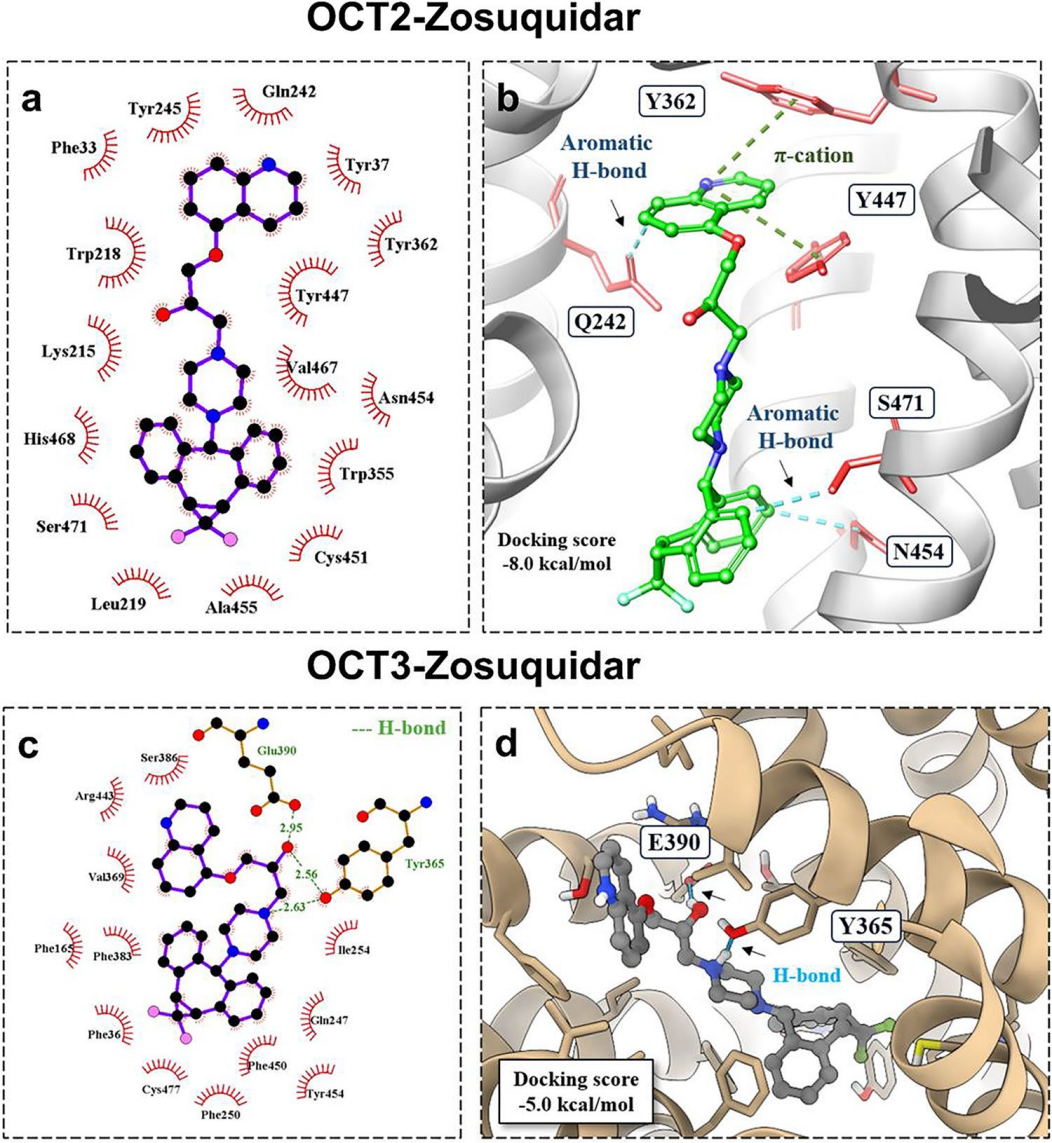
2D and 3D molecular interactions of zosuquidar with OCT2 and OCT3 transporters. **a** 2D interaction diagram of OCT2-zosuquidar, showing key amino acid residues around the compound. **b** 3D molecular model highlighting the interaction between zosuquidar (in green) and amino acids within OCT2, with specific interactions like aromatic hydrogen bonds and π-cation interactions annotated. **c** 2D interaction diagram for OCT3-zosuquidar, illustrating the involvement of different amino acid residues. **d** 3D molecular model for OCT3-zosuquidar, showing the compound in grey colour within its binding pocket on the transporter, emphasising an H-bond

For OCT2, zosuquidar did not form any hydrogen bond interactions, but the docking score was similar to OCT1 (Table 1), indicating mostly hydrophobic interactions (Fig. 3a). This weak interaction was extended with cation-pi interactions, a stabilising electrostatic interaction between residues Y362 and Y447, and ligand stabilisation with more aromatic hydrogen bonds in OCT2 (Fig. 3b).

Interestingly, OCT3 had two hydrogen bond interactions with E390 and Y365, both known inhibitor interaction sites with OCT3 (Fig. 3c) (Khanppnavar et al. 2022). However, the docking score of −5.0 kcal/mol (Table 1) suggests that these interactions do not favour the posing in the OCT3 transporter (Fig. 3d). In silico data confirm that OCT1 was more favourable for the binding of the zosuquidar inhibitor site compared to other transporters, based on their posing and noncovalent interactions.

## Discussion

Zosuquidar is a widely used and very potent experimental P-gp inhibitor, which is also commended to be very specific. Indeed, early experiments have demonstrated lack of significant inhibition of other ABC-transporters like MRP1, MRP2 or BCRP (Dantzig et al. 1999; Shepard et al. 2003). However, when using it as a control P-gp inhibitor in an experimental study, we obtained data suggesting that zosuquidar might also inhibit OCTs, questioning its selectivity. If zosuquidar also inhibited OCTs, this would interfere with assays using P-gp substrates that are also transported by OCTs, like it is the case for the often-used P-gp probe substrate rhodamine-123 (Jouan et al. 2014). We therefore investigated in vitro whether zosuquidar is able to inhibit the transport of the prototypical OCT substrate metformin in HEK293 cells overexpressing OCT1, OCT2, or OCT3. In our experimental settings, it most strongly inhibited metformin uptake through OCT1 with an IC_50_ value of 7.5 µM, followed by OCT3 and OCT2. Our data clearly demonstrate for the first time that zosuquidar indeed inhibits OCT1-3. For OCT1 this is in contrast to a previous study, in which 5 µM zosuquidar had no effect on the transport of the OCT1 substrate berberine (Nies et al. 2008), but this might be attributed to a different cell system, type of assay and substrate used. Moreover, at 5 µM, only a small part of OCT1 might be inhibited.

The in silico docking results correlate well with the in vitro inhibition data, confirming that zosuquidar has a higher binding affinity and inhibitory effect on OCT1 compared to OCT2 and OCT3 (Table 1). The detailed molecular interactions observed in the docking studies provide a mechanistic understanding of the differential inhibition observed in the in vitro assays.

Interestingly and important to consider, the inhibition potency for OCTs is much lower than that for most P-gp assays (≤ 0.1 µM) (Slapak et al. 2001; Green et al. 2001; Weiss and Haefeli 2006; Ozgür et al. 2018). Therefore, when using 1 µM zosuquidar or lower for P-gp inhibition, relevant inhibition of OCTs or OATP1A2 (IC_50_ = 6.7 µM) (Bakos et al. 2020) is largely ruled out. In contrast, when zosuquidar is used at 5 µM (e.g. Liu et al. 2014, 2017; Kort et al. 2015; Durmus et al. 2015) or 10 µM (e.g. Morad et al. 2017; Nielsen et al. 2019), bystander effects on other drug transporters should be considered when interpreting the data as we have previously observed (Bajraktari-Sylejmani et al. unpublished results).

In conclusion, we identified zosuquidar as a weak inhibitor of OCTs. However, this is of low experimental relevance as long as zosuquidar concentrations are kept below 1 µM for P-gp inhibition.

## Data Availability

All source data for this work (or generated in this study) are available upon reasonable request.
